# Association Between Metabolic Syndrome, Obesity, and Cognitive Performances in Individuals With Bipolar Disorders: Cross‐Sectional and Longitudinal Analyses in the FACE‐BD Cohort

**DOI:** 10.1111/acps.70048

**Published:** 2025-11-20

**Authors:** I. Palimaru, B. Etain, M. Leboyer, Y. Dansou, P. Favre, S. Gard, V. Aubin, F. Bellivier, R. Belzeaux, P. Courtet, C. Dubertret, E. Haffen, A. Lefrere, P. M. Llorca, E. Olié, M. Polosan, L. Samalin, R. Schwan, the FondaMental Academic Centers of Expertise in Bipolar Disorders (FACE‐BD) Collaborators, B. Etain, E. Olié, M. Leboyer, E. Haffen, P. M. Llorca, V. Barteau, S. Bensalem, O. Godin, H. Laouamri, K. Souryis, S. Hotier, A. Pelletier, F. Hergeta, J. Petrucci, L. Willaume, F. Bellivier, B. Etain, V. Hennion, E. Marlinge, J. Meheust, A. Richard, M. Carminati, H. Francisque, S. Gard, K. M’Bailara, C. Elkael, F. Hoorelbeke, I. Minois, J. Sportich, N. Da Ros, C. Dubertret, N. Mazer, C. Portalier, C. Scognamiglio, A. Bing, P. Laurent, L. Boukhobza, P. Courtet, S. Denat, B. Deffinis, D. Ducasse, M. Gachet, A. Lengvenyté, F. Molière, L. Nass, E. Olié, G. Tarquini, A. Lefrere, E. Moreau, J. Pastol, F. Groppi, H. Polomeni, J. Bauberg, L. Lescalier, I. Muraccioli, A. Suray, R. Cohen, J. P. Kahn, M. Milazzo, O. Wajsbrot‐Elgrabli, T. Bougerol, A. Pouchon, A. Bertrand, B. Fredembach, A. Suisse, Q. Denoual, M. Polosan, A. M. Galliot, L. Brehon, G. Bonny, L. Durand, V. Feuga, N. Kayser, P. Roux, V. Aubin, I. Cussac, M. A. Dupont, J. Loftus, I. Medecin, C. Dubertret, N. Mazer, P. Laurent, L. Samalin, P. M. Llorca, M. Mennetrier, T. Bonnet, D. Lacelle, M. Vayssié, C. Beal, O. Blanc, P. Roux, O. Godin

**Affiliations:** ^1^ Fondation FondaMental Créteil France; ^2^ Univ Paris Est Créteil, INSERM U955, IMRB, Team Translational NeuroPsychiatry Créteil France; ^3^ Université Paris Cité, INSERM UMR‐S 1144, Optimisation Thérapeutique en Neuropsychopharmacologie OTeN Paris France; ^4^ AP‐HP, Groupe Hospitalo‐Universitaire AP‐HP Nord, DMU Neurosciences, Hôpital Fernand Widal, Département de Psychiatrie et de Médecine Addictologique Paris France; ^5^ AP‐HP, Hôpitaux Universitaires Henri Mondor, Département Médico‐Universitaire de Psychiatrie et d'Addictologie (DMUIMPACT), Fédération Hospitalo‐Universitaire de Médecine de Précision en Psychiatrie (FHU ADAPT) Créteil France; ^6^ Neurospin, UNIACT, PsyBrain Team, CEA Paris‐Saclay Gif‐sur‐Yvette France; ^7^ Centre Hospitalier Charles Perrens, Pôle de Psychiatrie Générale et Universitaire Bordeaux France; ^8^ Pôle de Psychiatrie, Centre Hospitalier Princesse Grace Monaco Monaco; ^9^ IGF, Univ. Montpellier, CNRS, INSERM & Department of Psychiatry, CHU de Montpellier Montpellier France; ^10^ IGF, Univ. Montpellier, CNRS, INSERM, Department of Emergency Psychiatry and Acute Care, CHU Montpellier Montpellier France; ^11^ AP‐HP, Groupe Hospitalo‐Universitaire AP‐HP Nord, DMU ESPRIT, Service de Psychiatrie et Addictologie, Hôpital Louis Mourier Colombes France; ^12^ Université de Paris, Inserm UMR1266, Sorbonne Paris Cité, Faculté de Médecine Paris France; ^13^ Université de Franche‐Comté, UR 481 LINC, Service de Psychiatrie de l'Adulte, CIC‐1431 INSERM, CHU de Besançon Besançon France; ^14^ Pôle de Psychiatrie, Assistance Publique Hôpitaux de Marseille Marseille France; ^15^ INT‐UMR7289, CNRS Aix‐Marseille Université Marseille France; ^16^ Department of Psychiatry CHU Clermont‐Ferrand, University of Clermont Auvergne, CNRS, Clermont Auvergne INP, Institut Pascal (UMR 6602) Clermont‐Ferrand France; ^17^ Univ. Grenoble Alpes, Inserm, U1216, CHU Grenoble Alpes, Grenoble Institut Neurosciences Grenoble France; ^18^ Université de Lorraine, Centre Psychothérapique de Nancy, Inserm U1254 Nancy France; ^19^ Centre Hospitalier de Versailles, Service Universitaire de Psychiatrie d'Adulte et d'Addictologie Le Chesnay France; ^20^ Université Paris‐Saclay; Université de Versailles Saint‐Quentin‐En‐Yvelines; DisAP‐DevPsy‐CESP, INSERM UMR1018 Villejuif France

**Keywords:** bipolar disorder (BD), cognition, cognitive performances, longitudinal study, metabolic syndrome (MetS), neuropsychological test, obesity

## Abstract

**Introduction:**

Metabolic syndrome (MetS) has been suggested to be associated with cognitive impairments in bipolar disorder (BD); however, studies are limited by small sample sizes or cross‐sectional design. Our objective is to evaluate the cross‐sectional and longitudinal associations between MetS and cognitive performances in a large cohort of individuals with BD.

**Methods:**

1175 individuals with a DSM‐IV diagnosis of BD were included from the FACE‐BD cohort, assessed with a standardized battery of clinical and neuropsychological tests and followed up with a cognitive retest at 2 years for a subsample (*n* = 367). A global cognitive index was created by using a Principal Component Analysis. Associations between MetS and cognitive performances at baseline were explored using multiple analyses of covariance and linear mixed models were used for longitudinal data.

**Results:**

The prevalence of MetS was 21.5% in this sample. Multivariable analyses identified associations between MetS and poorer cognitive performance in the cross‐sectional analysis, independently of age, gender, education level, psychotropic treatments, and comorbidities. Specifically, individuals with MetS showed poorer results (global cognitive index, cognitive flexibility, inhibition, and verbal memory). After adjustment, the longitudinal analysis showed no change in the global cognitive index at year 2 and no time × metabolic syndrome interaction.

**Conclusions:**

Our results suggest that MetS is cross‐sectionally, but not longitudinally, associated with poorer cognitive performances in BD. This study highlights the importance of systematically and accurately screening for metabolic abnormalities in individuals with BD, and screening for cognitive deficit especially in individuals with MetS. Our results suggest that MetS is not a risk factor for cognitive decline during the follow‐up, but further longitudinal studies are required.

**Summary**

- Significant outcomes
  ○ Individuals with metabolic syndrome (MetS) showed poorer global cognitive index at baseline, including poorer cognitive flexibility, inhibition, and verbal memory
  ○ The findings from the cross‐sectional analysis suggest that managing metabolic syndrome may offer opportunities to improve cognitive function.
  ○ However, our results do not support a causal link from MetS to any cognitive decline during follow‐up.
- Limitations
  ○ We cannot exclude potential selection bias due to attrition even if individuals lost at follow‐up did not present specific characteristics.
  ○ Only baseline factors have been considered whereas factors modified during follow‐up could also have an impact on change in cognitive performances during follow‐up.
  ○ Our study did not include data on additional relevant variables such as physical exercise levels or dietary habits.

## 1 Introduction

Bipolar disorder (BD) is a chronic mood disorder affecting 1%–4% of the worldwide population [1]. A growing literature has repeatedly demonstrated that individuals with BD have a higher mortality rate, with a reduced life expectancy of almost 10–20 years, as compared to the general population [2]. This is mainly due to an increased prevalence of suicide, but also to the presence of somatic illnesses, particularly cardiovascular diseases, diabetes or cancers [3].

Metabolic Syndrome (MetS) is recognized as a leading cause of cardiovascular disease‐related mortality in the general population [4]. MetS is a cluster of five clinical and biological measures including abdominal obesity, hypertension, hypertriglyceridemia, decreased HDL‐cholesterol levels, and impaired glucose tolerance [5], predisposing individuals to diabetes, cardiovascular diseases, myocardial infarction, and stroke, but also to cognitive impairment and vascular dementia [6, 7]. According to a meta‐analysis published by Vancampfort et al., compared with general population groups, bipolar patients had higher metabolic syndrome rates (odds ratio = 1.98; 95% CI = 1.74–2.25) [8].

Cognitive impairment is a significant and often persistent feature of BD, affecting multiple domains such as attention, memory, executive functioning, or processing speed. Research findings indicate that 34%–60% of individuals with BD exhibit cognitive impairment during the euthymic phase [9, 10, 11, 12]. These impairments are observed during acute phases, but persist during euthymic phases, impacting daily functioning, occupational performance, and quality of life [12, 13, 14]. Understanding and addressing cognitive impairment is essential for comprehensive management, as it may contribute more to long‐term disability than mood symptoms alone. Given the significant functional impact of these cognitive impairments in BD, it is essential to identify risk factors they are associated with and potentially influenced by. Among these, metabolic abnormalities including obesity, dyslipidaemia, hypertension, insulin resistance as well as metabolic syndrome have been suggested [10, 15, 16].

A review published by Moreno and colleagues showed that working memory, verbal and declarative memory, attention, as well as executive and visuospatial functions were altered in individuals diagnosed with BD and a comorbid MetS [16]. Another article by Bora et al. reviewed the associations between obesity, MetS and its components and neurocognitive measures in BD. Overweight/obese individuals with BD have significantly more severe cognitive impairments compared with normal‐weighted ones. Some evidence for associations between cognition and other cardiovascular risk factors was observed, although relatively few studies have investigated the links between neurocognition and MetS, hypertension, diabetes, and dyslipidemia as compared to studies investigating only obesity [15]. Indeed, half of the studies reviewed focused on obesity and/or Body Mass Index (BMI) only without including MetS and its components, pointing to a significant gap in the literature. More recently, in a large sample of 966 individuals with BD, Ringin and colleagues showed that among traditional cardiometabolic risk factors, only blood pressure was associated with cognitive functioning in BD [10]. In addition to a literature mostly focusing on obesity and/or BMI, other limitations included small sample sizes, limited investigations of potential confounding factors, or the absence of longitudinal data.

Assessing metabolic syndrome offers a comprehensive perspective on the cumulative impact of interconnected metabolic abnormalities on cognitive function. Given the importance of MetS and cognitive impairment in the prognosis of BD, it is relevant to further explore their potential links and to address some of the gaps in the literature that are (1) the use of a large sample size to achieve reasonable power in a cross‐sectional study, (2) the disentanglement of the associations between MetS and obesity and different cognitive domains and (3) the longitudinal effects of MetS on cognition. Furthermore, examining each component individually (beyond the sole presence/absence of MetS or obesity) such as hypertension, dyslipidemia, hyperglycemia, and abdominal obesity, may yield important insights, as their effects may vary in both magnitude and underlying mechanisms. This combined approach enables a more nuanced understanding by capturing both the synergistic influence of the syndrome and the distinct contributions of its individual elements to cognitive outcomes.

### 1.1 Aim of the Study

Therefore, we first investigated the associations between the presence of MetS, all its components and obesity with cognitive performances in 1175 individuals assessed at baseline. Then, in a subsample of 528 individuals with follow‐up data, we performed a longitudinal study to explore the link between the presence of MetS at baseline and any potential cognitive decline over 2 years.

## 2 Materials and Methods

### 2.1 Study Population

Participants were recruited as part of the FondaMental Advanced Center of Expertise (FACE‐BD) cohort, coordinated by the non‐profit foundation FondaMental. At baseline, a multidisciplinary team (psychiatrists, (neuro)‐psychologists) interviewed the individuals using the SCID [17] and systematically recorded information related to the individuals' education, onset and course of BD and psychiatric comorbidities (anxiety and substance use disorders). Socio‐demographic data including age, gender, years of education, and information on individuals' current mood state, overall functioning, sleep quality, medications, lifetime number of mood episodes, hospitalizations and suicide attempts were recorded. Mood state was evaluated using the Montgomery and Åsberg Depression Rating Scale (MADRS) [18] and the Young Mania Rating Scale (YMRS) [19]; global functioning was assessed using the Functioning Assessment Short Test (FAST) [20] and sleep quality was measured using the Pittsburgh Sleep Quality Index (PSQI), in the validated French version [21]. A physical examination including a routine blood sample is performed to measure standard biological markers including complete blood count, blood glucose, lipid profile, Body Mass Index (BMI) with height and weight, waist circumference, and blood pressure. Individuals are evaluated at inclusion, followed up at 1 year and 2 years, during which they are re‐evaluated using the same protocol. A neuropsychological evaluation is proposed at baseline and 2 years.

The study included adult outpatients with BD type I, II, or not otherwise specified (NOS) according to DSM‐IV criteria. All patients were euthymic at the time of inclusion and we selected, for this study, only participants with a MADRS score ≤ 10 and a YMRS score ≤ 12, as well as those who benefited from neuropsychological examinations at baseline. Individuals with a history of neurological or sensory disorders (stroke, epilepsy, multiple sclerosis), dyslexia, dysorthographia, dyscalculia, dysphasia, dyspraxia, language delay, substance‐related disorders within the past month, or electroconvulsive therapy within the past year were also excluded from the study (Flowchart on Figure S1).

The institutional review board (Comité de Protection des Personnes Ile de France IX; January 18, 2010) approved the assessment protocol, in compliance with French regulations for non‐interventional studies. As requested by the institutional review board, an informational letter was given to all participants. A non‐opposition form was signed by all participants as this study include data coming from regular healthcare assessments.

### 2.2 Neurocognitive Battery Test

To assess cognitive performance of the participants, we used a battery of neuropsychological tests that explore several domains of cognitive skills, at the first visit and at 2 years of follow‐up. The tests were administered in a pre‐established order by experienced neuropsychologists, and the entire testing process took 120 min, including short breaks between each test.

The standardized test battery followed the recommendations of the International Society for Bipolar Disorders [22], and consisted of 16 tests assessing 5 main cognitive domains, from which we retained the raw values.

1. *Attention*: inattentiveness (Continuous Performance Test (CPT): omission + commission) [23].
2. *Executive function*: cognitive flexibility (Trail Making Test (TMT): subtest B) [24], inhibition (Stroop part C: number of ink colours named in 45 s) [25], and verbal fluency (Test of verbal fluency: total number of words with P letters and total number of correct word (animals)) [26].
3. *Processing speed*: Wechsler Adult Intelligence Scale (WAIS) version III/IV [27]: digit symbol‐coding, TMT‐A [24], Stroop test (Part A: number of words read in 45 s; Part B: number of colours named in 45 s) [25].
4. *Working memory*: Digit span memory test [27].
5. *Verbal memory*: California Verbal Learning Test (CVLT): Trials 1–5 free recall total correct, short‐delay free recall, short‐delay cued recall, long‐delay free recall, long‐delay cued recall [28].

We categorized the tests according to the cognitive domain framework used in previous studies on BD [29, 30].

To create a global cognitive index, a principal component analysis (PCA) with parameters set for “no rotation” and Eigenvalues > 1.0 was performed including all tests above. We generated *z*‐scores for each test included in the Principal Component Analysis (PCA) by using the formula *z* = (*x* − *μ*)/*σ*, where *x* represents the raw score of the individual participant, μ is the population mean (i.e., the mean of the entire sample), and *σ* is the population standard deviation. We then retained the first computed factor as a general cognitive ability measure called the “Global Cognitive Index” (GCI) [31]. Only tests with measures loading substantially onto the first component, that is, a factor loading above absolute 0.35 have been included in the PCA analysis, meaning all tests listed in this section, except the two CPT tests for which the factor loading was below 0.35. GCI, as the first component retained by the PCA, explained 43% of the variance (Table S1, Table S2, Figure S2). Given the loadings, the higher the GCI was, the higher the performances. A significant decrease during the follow‐up can be viewed as a “cognitive decline”. Sensitivity analyses including the two additional CPT tests into the PCA did not change the association between metabolic syndrome and global cognitive index (data not shown).

### 2.3 Metabolic Syndrome Definition

Blood samples were taken in the morning after fasting for at least 8 h. Two sitting blood pressure readings were taken automatically 30 s apart after a 5 min rest. A third was added if values differed by > 10 mmHg, and the average of the two closest was recorded in the database. Individuals with BD were considered to have MetS if they met at least three of the five following criteria, based on the modified National Cholesterol Education Program Adult Treatment Panel III [32]: abdominal obesity (waist circumference > 94 cm for men and > 80 cm for women), low levels of high‐density lipoprotein (HDL) cholesterol (< 1.03 mmol/L in men and < 1.3 mmol/L in women or specific treatment), hypertriglyceridemia (≥ 1.7 mmol/L or lipid‐lowering medication), high fasting glucose concentration (≥ 5.6 mmol/L or taking glucose‐lowering medication), and high blood pressure (≥ 130/85 mmHg or on antihypertensive medication).

### 2.4 Statistical Analyses

Categorical variables were presented as percentages and quantitative variables as means (standard deviation). The sociodemographic and clinical characteristics, as well as cognitive performance of individuals with or without MetS were compared using chi‐square tests for categorical variables and Student's or Mann–Whitney tests (depending on the distribution of the variables) for continuous variables. We estimated associated crude odds ratios (ORs) with 95% CIs (Confidence Intervals) and chi‐square values, and degrees of freedom were also presented.

To investigate the associations between MetS, obesity and MetS components with cognitive performances, we performed two multivariate analyses of covariance adjusting for: (1) gender, age, and level of education and (2) additionally on potential confounders that were: gender, age, education level, lifetime cannabis and alcohol disorders, smoking status, psychotropic treatment (anxiolytics, hypnotics, antipsychotics, lithium, anticonvulsant and antidepressant) and sleep disorders. Estimated means, F‐value, and Cohen's effect size were presented. Separate tests were performed for Global Cognitive Index, as well as for each neuropsychological test. Associations between the Global Cognitive Index and each MetS component, as well as body mass index, were also performed.

For the longitudinal analyses, we used a linear mixed model to deal with loss to follow‐up and handle repeated measures. Analyses have been adjusted for age, gender and education level. A false discovery rate (FDR) correction has been applied to account for multiple testing. Statistical analyses were conducted using the statistical software SPSS Statistics 28.0.

## 3 Results

After applying inclusion and exclusion criteria, a total of 1175 individuals with data about MetS and cognitive performances can be included in the analyses. Mean age of participants was 39.7 (SD = 13.0) years, 62% were females, 52.85% had BD type I and mean duration of illness was 16.7 (SD = 12.4) years.

### 3.1 Prevalence of Metabolic Syndrome and Associated Factors

As shown in Table 1, the prevalence of MetS in the sample was 21.5% and was significantly higher in men than in women (26.17% and 18.54%, respectively, *p* = 0.002). We observed that 96.0% of individuals with MetS had abdominal obesity, 76.2% had high blood pressure, 66.7% had hypertriglyceridemia, 63.9% had low High‐Density Level (HDL) cholesterol, and 45.2% had high fasting glucose concentrations. Factors associated with MetS are shown in Table 2. The risk of MetS was significantly higher in men compared to women (OR = 1.56 (1.17–2.06)) and in older participants (OR = 1.05 (1.03–1.06)). Individuals with MetS also had a higher frequency of sleep disturbances compared to those without MetS (52.5% vs. 43.8%; OR = 1.42 (1.01–1.10)), higher BMI as expected, and a trend to more frequent current tobacco daily use. By contrast, the frequency of lifetime cannabis use disorder was lower in individuals with MetS (13.38% vs. 22.6%; OR = 0.53 (0.33–0.86)). No association between MetS and BD subtype, number or type of mood episodes, rapid cycling or suicide attempt was observed. Regarding psychotropic medications, only individuals using anxiolytics medication at baseline had a higher risk of MetS (OR = 1.65 (1.12–2.43)), whereas we did not find any association between MetS and the use of neuroleptics, antidepressants, lithium, anticonvulsants and antipsychotics (Table 1).

**TABLE 1:** Univariable associations between metabolic syndrome and socio‐demographic variables, clinical variables and medication in individuals with BD.

	Metabolic syndrome at baseline		Df	*X* ^2^	*p* ^a^ FDR	OR CI 95%^b^
	No	Yes				
	(*N* = 923, 78.5%)	(*N* = 252, 21.5%)				
*Socio‐demographic characteristics*						
Gender, *n* (%)						
Women	593 (81.5)	135 (18.5)	1	9.57	0.018	1 (ref)
Men	330 (73.8)	117 (26.2)				1.56 (1.17–2.06)
Age, mean (SD)	38.0 (12.6)	46.2 (12.6)		78.2	< 0.001	1.05 (1.03–1.06)
Education level, mean (SD)	14.6 (2.6)	14.1 (3.1)		7.47	0.027	0.93 (0.89–0.98)
*Clinical characteristics*						
Age at BD onset, mean (SD)	23.8 0 (8.8)	26.40 (9.8)		13.6	< 0.001	1.03 (1.01–1.04)
Number of mood episodes, mean (SD)	7.6 (8.5)	8.48 (9.7)			0.25	1.01 (0.99–1.03)
History of rapid cycling, *n* (%)			1	0.12		
No	715 (88.3)	95 (86.5)			0.73	1 (ref)
Yes	179 (11.7)	28 (13.5)				0.75 (0.75–1.75)
History of suicide attempt, *n* (%)	291 (32.1)	86 (36.0)	1	1.30	0.33	1.19 (0.88–1.60)
Current depressive symptoms, mean (SD)	4.05 (3.2)	4.22 (3.1)		0.73	0.39	1.02 (0.97–1.06)
Current manic symptoms, mean (SD)	1.83 (2.8)	2.03 (2.9)		1.22	0.31	1.02 (0.98–1.07)
Diagnosis, *n* (%)						
BD Type 1	478 (77.0)	143 (23.0)	2	2.12		1 (ref)
BD Type 2	367 (80.7)	88 (19.3)			0.42	0.83 (0.66–1.06)
BD not specified	78 (78.8)	21 (21.2)			0.57	0.92 (0.61–1.38)
*Comorbidities*						
Lifetime tobacco status, *n* (%)						
No	427 (80.9)	101 (19.1)	1	1.71	0.31	1 (ref)
Current smokers	469 (76.6)	143 (23.4)				1.29 (0.97–1.72)
Lifetime alcohol use disorder, *n* (%)						
No	738 (79.1)	195 (20.9)	1	0.50	0.51	1 (ref)
Yes	135 (76.7)	41 (23.3)				1.15 (0.78–1.69)
Lifetime cannabis use disorder, *n* (%)						
No	737 (77.4)	215 (22.6)	1	6.8	0.04	1 (ref)
Yes	136 (86.6)	21 (13.4)				0.53 (0.33–0.86)
Body Mass Index, *n* (%)						
< 30	818 (84.8)	147 (15.2)	1	142.9	< 0.01	1 (ref)
≥ 30	85 (45.5)	102 (54.5)				6.68 (4.77–9.36)
Sleep disturbances (PSQI ≥ 5), *n* (%)	116 (43.8)	128 (52.5)	1	4.0	0.13	1.42 (1.07–1.88)
*Psychotropic medications, n (%)*						
Antidepressants	233 (74.92)	78 (25.08)	1	2.09	0.14	1.27 (0.91–1.75)
Anxiolytics	109 (69.87)	47 (30.13)	1	6.69	0.04	1.65 (1.12–2.43)
Hypnotics	65 (74.71)	22 (25.29)	1	0.48	0.51	1.20 (0.72–2.00)
First generation antipsychotics	253 (80.57)	18 (28.57)	1	1.52	0.31	1.43 (0.80–2.53)
Second generation antipsychotics	262 (74.86)	88 (25.14)	1	2.63	0.23	1.30 (0.94–1.79)
Lithium	253 (80.57)	61 (19.43)	1	2.33	0.25	0.76 (0.54–1.08)
Anticonvulsants	350 (75.11)	116 (24.89)	1	3.70	0.13	1.36 (0.99–1.87)

Abbreviations: BD, bipolar disorder; PSQI, Pittsburgh Sleep Quality Index.

^a^ χ^2^ test for categorical variables and Student's or Mann–Whitney/Kruskal–Wallis tests for continuous variables.

^b^ Logistic regression analysis, Odds Ratios [OR] 95% Confidence Interval [CI].

**TABLE 2:** Association between metabolic syndrome and cognitive performances at baseline in individuals with BD.

	Metabolic syndrome at baseline		Effect size (*d*)	Multivariable	Multivariable
	No	Yes		*p* ^a^	*p* ^b^
	(*N* = 923, 78.5%)	(*N* = 252, 21.5%)		FDR	FDR
*Cognitive performances (estimated means (SE))*					
Global Cognitive Index	0.03 (0.02)	−0.23 (0.05)	0.31	**< 0.001**	**0.03**
*Processing speed*					
WAIS‐III/IV: digit symbol‐coding (nb of correct symbols)	33.9 (0.24)	32.5 (0.47)	0.18	**0.02**	0.09
TMT‐A: time in sec	32.2 (0.4)	32.8 (0.7)	−0.05	0.49	0.90
STROOP Test: Part A (nb of words read in 45 s)	105 (0.53)	101 (0.99)	0.23	**0.008**	**0.002**
STROOP Test: Part B (nb of colors named in 45 s)	73.0 (0.43)	70.9 (0.82)	0.17	**0.03**	**0.04**
*Attention*					
CPT‐II (nb of words: omissions)	7.2 (0.80)	10.1 (1.48)	−0.15	0.09	0.05
CPT‐II (nb of words: commissions)	12.0 (0.32)	13.2 (0.59)	−0.14	0.10	0.64
*Executive function*					
STROOP Test, Part C (nb of ink colors in 45 s)	44.4 (0.34)	41.9 (0.65)	0.25	**0.005**	**0.001**
TMT‐B: time in sec	75.0 (1.17)	83.8 (2.24)	−0.25	**0.005**	**0.02**
Verbal Fluency: nb of words with the letter P	24.5 (0.31)	23.6 (0.60)	0.10	0.19	0.52
Verbal Fluency: nb of animals named in 1 min	32.9 (0.28)	31.0 (0.55)	0.23	**0.02**	**0.05**
*Verbal memory*					
CVLT: nb of correct words					
Trials 1–5 free recall total correct	57.9 (0.33)	56.3 (0.62)	0.17	**0.02**	**0.03**
Short‐delay free recall	12.1 (0.09)	11.4 (0.18)	0.22	**0.003**	**0.01**
Short‐delay cued recall	12.6 (0.09)	12.1 (0.17)	0.19	**0.01**	**0.02**
Long‐delay free recall	12.8 (0.08)	12.4 (0.16)	0.16	**0.03**	**0.04**
Long‐delay cued recall	13.0 (0.09)	12.6 (0.17)	0.16	**0.02**	0.09
*Working memory*					
WAIS‐III: Digit span subtest	23.9 (0.23)	23.0 (0.45)	0.13	0.08	0.26

*Note*: In bold: *p* values < 0.05 after correction (FDR: false discovery rate).

Abbreviations: CPT, continuous performance task; CVLT, California verbal learning test; *d*, Cohen's *d*; nb, number; TMT, trail making test; WAIS‐III, Weschler Adult Intelligence Scale‐III.

^a^ Analysis of covariance adjusted for sex, age and education level.

^b^ Analysis of covariance adjusted for sex, age, education level, lifetime cannabis and alcohol use disorders, smoking status, sleep disorders, and current psychotropic medications (anxiolytics, hypnotics, antipsychotics, lithium, anticonvulsants, and antidepressant).

### 3.2 Cross‐Sectional Analyses

Table 2 summarizes the association between MetS and cognitive performance at baseline for the GCI as well as for all cognitive measures classified by domains. We adjusted for the following potential confounders: gender, age, education level, lifetime cannabis disorders, tobacco status, sleep disturbances, and psychotropic treatments. All psychotropic treatments were included in the model given their potential associations with cognitive performance (Table S3). Individuals with MetS at baseline had a lower global cognitive index (i.e., meaning lower cognitive ability) compared to individuals without MetS at baseline (−0.23 (SE = 0.05) vs. 0.03 (SE = 0.02), *p* < 0.01). When each cognitive test was investigated separately, analyses showed that individuals with MetS had lower cognitive performance on cognitive flexibility (TMT part B), inhibition (Stroop part C), verbal memory (all CVLT measures, excepting long‐delay cued recall), and processing speed (Stroop test, part A and B). No significant association for working memory and for attention was observed.

**FIGURE 1:**
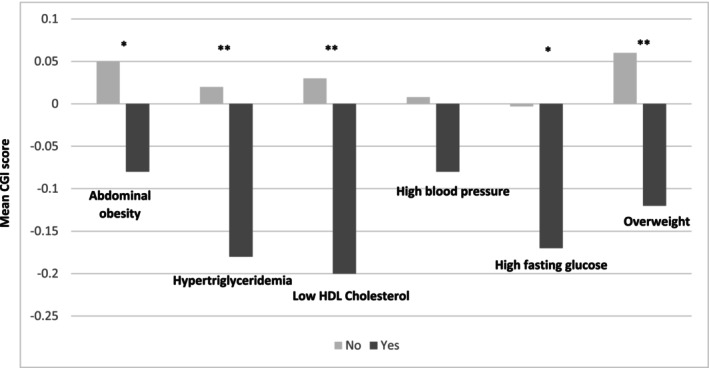
Comparison between each MetS component, body mass index, and Global cognitive Index (GCI) score at baseline. Estimated Mean CGI score. * Analysis of covariance adjusted for sex, age and education level. ** Analysis of covariance adjusted for metabolic syndrome, sex, age, education level, lifetime cannabis, tobacco and alcohol disorders, smoking status, sleep disorders, psychotropic treatment (anxiolytics, hypnotics, antipsychotics, lithium, mood stabilizers). HDL‐cholesterol, high density level‐cholesterol.

Figure 1 and Table S4 illustrate the multivariable associations between the Global Cognitive Index and each component of the MetS, as well as with body mass index. After adjusting for all potential confounders, a lower Global Cognitive Index was observed in individuals presenting hypertriglyceridemia, low‐HDL cholesterol levels and overweight. No association was found with abdominal obesity, high fasting glucose levels and hypertension.

### 3.3 Longitudinal Analyses

Among the 1175 individuals included at baseline, 528 participants (44.9%) attended the follow‐up visit at year 2 and 367 had information to evaluate the cognitive performances. Among them, 75 (20.4%) had MetS at baseline.

Characteristics of participants with or without follow‐up are presented in Table S5. Data are not described in detail in the text. Nevertheless, there was no significant difference between the two groups (lost or not lost at follow‐up) regarding the presence of MetS, BMI nor cognitive impairment (evaluated using the Global Cognitive Index).

The longitudinal associations between MetS at baseline and the evolution of cognitive performances were shown in Table 3. After taking into account gender, age and education levels, results from the linear mixed model showed that the Global Cognitive Index did not change significantly between baseline and 24 months. Regarding the different domains of cognitive functioning, all performances in working and verbal memory significantly improved over the 2‐year period. Results were more contrasted for other domains such as attention, processing speed and executive function, that is, performances in some tests improved while others did not change. There was no time × MetS interaction on the Global Cognitive Index or on any cognitive tests.

**TABLE 3:** Longitudinal data: Association between baseline Mets and evolution of cognitive performances in individuals with BD during the follow‐up.

		Estimate (*β*) (SE)	df	*T* value	*p* ^a^
	*Global cognitive index*				
	Time (in month)	−0.0012 (0.0017)	329.6	−0.937	0.349
	Time × Metabolic syndrome	−0.0029 (0.0038)	332.5	−0.762	0.447
Processing speed	*WAIS‐III/IV: digit symbol‐coding (nb of correct symbol)*				
	Time (in month)	0.057 (0.02)	577.5	3.569	0.0004
	Time × Metabolic syndrome	0.045 (0.04)	583.0	1.286	0.199
	*TMT‐A: time in sec*				
	Time (in month)	−0.115 (0.02)	519.2	−4.845	< 0.0001
	Time × Metabolic syndrome	0.029 (0.05)	525.3	0.551	0.582
	*STROOP Test: Part A (nb of words read in 45 s)*				
	Time (in month)	0.045 (0.02)	431.0	0.167	0.867
	Time × Metabolic syndrome	−0.111 (0.06)	436.4	−1.865	0.063
	*STROOP Test: Part B (nb of colors named in 45 s)*				
	Time (in month)	0.0039 (0.024)	448.4	0.163	0.870
	Time × Metabolic syndrome	0.028 (0.053)	455.9	0.527	0.599
Attention	*CPT‐II (nb of word: omissions)*				
	Time (in month)	−0.012 (0.06)	478.3	−0.200	0.841
	Time × Metabolic syndrome	0.059 (0.13)	502.3	0.441	0.659
	*CPT‐II (nb of word: commissions)*				
	Time (in month)	−0.05 (0.02)	510.9	−2.655	0.008
	Time × Metabolic syndrome	−0.06 (0.05)	523.3	−1.421	0.156
Executive function	*STROOP Test: Part C (nb of ink colors in 45 s)*				
	Time (in month)	0.024 (0.018)	429.0	2.326	0.020
	Time × Metabolic syndrome	−0.003 (0.039)	434.9	−0.008	0.993
	*TMT‐B: time in sec*				
	Time (in month)	−0.05 (0.07)	565.8	−0.679	0.498
	Time × Metabolic syndrome	−0.289 (0.16)	577.9	−1.774	0.076
	*Verbal Fluency: nb of words with letter P*				
	Time (in month)	0.023 (0.02)	992.8	1.031	0.303
	Time × Metabolic syndrome	−0.022 (0.05)	1012.8	−0.435	0.663
	*Verbal Fluency: nb of animals named in 1 min*				
	Time (in month)	0.053 (0.02)	506.9	3.057	0.002
	Time × Metabolic syndrome	−0.012 (0.04)	516.4	−0.319	0.749
Verbal memory	*CVLT: nb of correct words*				
	Time (in month)	0.141 (0.02)	495.9	7.337	< 0.0001
	Time × Metabolic syndrome	−0.035 (0.04)	494.1	−0.826	0.409
	*Trials 1–5 free recall total correct*				

	Time (in month)	0.041 (0.005)	512.5	7.014	< 0.0001
	Time × Metabolic syndrome	−0.011 (0.013)	510.9	−0877	0.381
	*Short‐delay free recall*				
	Time (in month)	0.032 (0.0053)	477.6	6.086	< 0.0001
	Time × Metabolic syndrome	0.0056 (0.011)	475.8	0.484	0.628
	*Short delay cued recall*				
	Time (in month)	0.033 (0.005)	496.9	6.683	< 0.0001
	Time × Metabolic syndrome	−0.021 (0.01)	495.1	−1.875	0.061
	*Long‐delay free recall*				
	Time (in month)	0.031 (0.0054)	514.1	5.799	< 0.0001
	Time × Metabolic syndrome	0.0045 (0.019)	512.5	0.378	0.705
	*Long‐delay cued recall*				
	Time (in month)	0.041 (0.01)	438.0	3.691	0.0002
Time × Metabolic syndrome	−0.023 (0.025)	441.4	−0.949	0.343
Working memory	*WAIS‐III: Digit span subtest*				
	Time (in month)	0.041 (0.011)	438.0	3.691	0.0002
	Time × Metabolic syndrome	−0.023 (0.025)	441.4	−0.949	0.343

Abbreviations: CPT, continuous performance task; CVLT, California verbal learning test; nb, number; TMT, trail making test; WAIS‐III/IV, Weschler Adult Intelligence Scale‐III/IV.

^a^ *p* Value corrected for age, gender, education level.

## 4 Discussion

The main results of this study can be summarized as follows: MetS is cross‐sectionally associated with poor global cognitive performance in individuals with BD, especially in global cognitive ability, verbal memory, executive function (cognitive flexibility and inhibition) and processing speed. During the follow‐up, cognitive performance remains stable or improves for some. No time *×* MetS interaction is observed in the longitudinal analyses; therefore, MetS at baseline is unlikely to predict any cognitive decline at year 2 in this sample.

We found a prevalence of MetS of 21.5% in the FACE‐BD cohort. Earlier research examining the presence of MetS in individuals with BD has indicated significant disparities in the prevalence rates, encompassing a range of 54.2% to 67% [33, 34]. The lower prevalence of MetS in France, could be attributed to various factors, including the definition used, and characteristics of the participants including age of participant, education level, BD severity or coexisting health and psychiatric comorbidities.

The results of the cross‐sectional analyses are consistent with the two previous meta‐analyses and reviews reporting lower cognitive performances in individuals with BD with and metabolic syndrome [15, 16]. In a study including 143 individuals with BD, those with MetS experienced lower overall functioning and more significant impairments in executive functions (evaluated with the Wisconsin Card Sorting Test) [35]. Similar findings were observed in another cross‐sectional study comparing 148 euthymic individuals with BD and 117 healthy controls from the BIPFAT/BIPLONG study [36]. In addition, Beunders et al. found an association between MetS and poorer verbal fluency performances, in a sample of 125 individuals with older‐age BD [37]. Investigating BMI and MetS components separately, we found associations between lipid disturbances, overweight and global cognitive functioning. Similar results have been previously observed in the literature: in a sample of 57 individuals (including 23 with BD), individuals with poorer cognitive flexibility presented higher triglycerides plasmatic levels [38]. Mazza et al. suggested an association between BMI and the brain fiber tracts present in networks that play a crucial role in neurocognitive functioning and also a correlation between triglycerides, cholesterol and markers of oxidative stress [39]. On the contrary, a recent large study investigating different physiological risk factors including alterations in lipids and glucose found no association with cognition [10]. In our study, no significant association between lower global cognition and high blood pressure has been found which is not consistent with some previous studies [10, 40, 41]. Our results on BMI are also consistent with those from previous studies. In the meta‐analysis from Bora et al., authors suggest that individuals with BD and with overweight/obesity displayed poorer performances, especially in tasks involving executive functions and processing speed with the preservation of other cognitive domains [15].

Regarding the longitudinal analyses, there was no association between MetS at baseline and cognitive decline. This is consistent with the results from the only longitudinal study published so far, in a cohort of 57 individuals with BD [42] followed up 1 year. On the opposite, a longitudinal study, not studying metabolic syndrome but obesity showed a significant association between obesity and lower cognitive performance over 6 years of follow‐up [43]. In addition, another longitudinal study including 38 euthymic individuals with BD followed up 12 months, found that high baseline BMI predicted a decrease in working memory performance [44].

The cross‐sectional association between MetS and cognitive impairment might be explained by several hypotheses. MetS is linked to vascular abnormalities, especially caused by high blood pressure and dyslipidaemia, leading to atherosclerosis, which can be responsible for reduced blood flow and oxygenation to the brain [45], leading to micro‐ and/or macro‐vascular changes in the brain. Additionally, MetS and obesity are metabolic states that trigger chronic low‐grade inflammation, increasing oxidative stress and insulin resistance [46, 47] which can be responsible for changes in the connectivity in cerebral areas responsible for cognitive functions [48]. Inflammatory processes (e.g., microglia activation and elevated cytokine levels) could therefore disrupt neurobiological mechanisms regulating cognition, including homeostatic plasticity, neurogenesis, neurotrophic factor, the HPA and the kynurenine pathways [49]. It is important to note, that many of the biological pathways mentioned here are also implicated in BD per se, making it difficult to attribute the observed effects exclusively to metabolic syndrome. This suggests that a cumulative or interactive effect of both BD and MetS on cognitive impairment may be involved. Furthermore, obesity can be associated with obstructive sleep apnea syndrome, which in turn can negatively impact cognitive function [50]. Many other factors could contribute both to cognitive impairment and to MetS in individuals with BD, including history of childhood trauma [51], lifestyle factors (unhealthy diet, low physical activities, addictions and sleep disorders) or the use of some medications used in BD—such as antipsychotics, valproate or benzodiazepines [52, 53]. Our results are concordant with the literature data showing an association between psychotropic drugs intake and lower global cognitive performances.

### 4.1 Strengths

This study has several strengths, including a large sample size, the use of consistent and comprehensive standardized diagnostic protocols and neuropsychological assessments. We excluded individuals who exhibited even mild symptoms of depressive or (hypo)manic phase, as they could potentially interfere with the interpretation of the results. Additionally, individuals with a substance use disorder in the month prior to inclusion were excluded for the same reasons. Our analyses also considered a large number of potential confounding factors and applied stringent criteria for inclusion. Moreover, given that the literature investigating longitudinal associations between obesity and cognitive impairment in BD is scarce, this is the first study, to our knowledge, evaluating the longitudinal association between MetS, its components and cognitive impairment in individuals with BD.

### 4.2 Limitations

However, some limitations should also be discussed including the significant number of participants lost to follow‐up which may affect the reliability and generalizability of the longitudinal findings. To address this issue, we conducted a comparison of characteristics between participants with or without data at follow‐up, and we did not find any significant differences between the two groups regarding our major outcomes of interest (MetS and cognitive functioning). However, we cannot exclude potential selection bias due to attrition. Other limitations are the relatively small number of patients (*n* = 528) followed up as well as the time of follow‐up duration that might be too short to observe any change in cognitive functioning. Longer follow‐up periods would be more suitable to capture potential delayed effects of MetS on cognition. In addition, it should be noted that our results apply only to individuals who fulfilled the stringent inclusion and exclusion criteria and may not be generalized to all individuals with BD. For instance, those with more depressive symptoms at baseline might be more likely to be those with both cognitive deficits and MetS criteria. Finally, we only consider baseline factors whereas factors modified during follow‐up such as medication intake or dosage could also have an impact on cognition change in the follow‐up. Even if our analyses were adjusted for sleep quality, we do not have analyzed the incidence of obstructive sleep apnoea syndrome (OSAS) in our cohort whereas evidence suggested that OSAS can lead to a decline in cognitive functions or even lead to permanent brain damage [50]. Moreover, our study did not include data on other relevant variables such as physical exercise levels or dietary habits.

## 5 Conclusion

We observed a cross‐sectional association between MetS and lower cognitive performance among individuals with BD, particularly for processing speed, verbal memory, and executive functions (cognitive flexibility and inhibition). However, the longitudinal analysis did not show any association between MetS and cognitive impairment in individuals with BD.

This study opens new perspectives and could serve as a starting point for future research. From a clinical point of view, these findings might have potential implications for the management of individuals with BD. First, individuals with BD and MetS had worse cognitive performances at baseline. This may be an indirect indicator for the indication of a cognitive assessment. Even if our results did not identify any time × MetS interaction on changes in cognitive performances, we cannot exclude that improving metabolic health would not improve cognition. Indeed, in the general population, evidence suggests that weight‐reduction programs can improve cognition [54]. Similar trials should be considered to be designed for BD to test the effectiveness of a similar approach in improving cognition in these individuals. In addition, although current knowledge is limited, some studies investigating the potential effect of semaglutide, a well‐known GLP‐1 agonist, on weight loss also described the protective effect of semaglutide in the context of depression. Animal models and clinical studies suggested that semaglutide might decrease hippocampal neuroinflammation, promote neurogenesis via the insulin/GLP‐1 pathway, and modulate the gut microbiota [55]. In a retrospective cohort study using electronic health records and covering more than 100 million patients in the USA, De Giorgi et al. suggested that semaglutide use could extend beyond managing diabetes, potentially offering unexpected benefits in the treatment and prevention of cognitive decline (De Giorgi et al. 2024). Moreover, a healthy diet might have a positive impact on cognition in individuals with schizophrenia [56].

Our work addresses a significant gap in the literature regarding the longitudinal association between MetS and cognition in BD. Even if our longitudinal analyses do not support a causal association between MetS and cognitive decline, the findings from our cross‐sectional analysis suggest that managing metabolic syndrome may offer opportunities to improve cognitive function.

## Author Contributions

O. Godin and B. Etain conceived and designed the study, supervised the project, and revised the manuscript critically for important intellectual content. I. Palimaru and Y. Dansou analyzed and interpreted the data, created the figures and statistical models. I. Palimaru and O. Godin drafted the initial version of the manuscript. B. Etain, L. Leboyer, P. Favre, Aubin V., Bellivier F., Belzeaux R., Courtet P., Dubertret C., Haffen E., Lefrere A., Llorca P. M., Olié E., Polosan M., Samalin L., Schwan R, P. Roux contributed to data collection, data interpretation and provided critical revisions of the manuscript. All authors read and approved the final manuscript.

## Disclosure

The authors have nothing to report.

## Ethics Statement

The authors assert that all procedures contributing to this work comply with the ethical standards of the relevant national and institutional committees on human experimentation and with the Helsinki Declaration of 1975, as revised in 2008.

## Conflicts of Interest

The authors declare no conflicts of interest.

## Supporting information


**FIGURE S1:** Flowchart showing the selection of participants from the FACE‐BD cohort.
**FIGURE S2:** Scree plot of Eigenvalues.
**TABLE S1:** Result of the PCA: Eigenvalues of the correlation Matrix.
**TABLE S2:** Results from PCA: Factor Pattern.
**TABLE S3:** Association between global cognitive index and psychotropic medications in individuals with BD at baseline.
**TABLE S4:** Association between global cognitive index, body mass index and MetS components.
**TABLE S5:** Comparison between individuals with BD with a follow‐up visit and individuals with BD lost during follow‐up (univariate analysis).

## Data Availability

Due to ethical and legal restrictions, data involving clinical participants cannot be made publicly available. All relevant data is available upon request to the Fondation FondaMental for researchers who meet the criteria for access to confidential data. A charter describing data access, rules (composition of the submission file, evaluation of the request, and transmission of the data) and publication policies (authorship, acknowledgements) has been established and is available on our web site (www.fondation‐fondamental.org). An external request from academic labs who are not members of the FondaMental network, requires a Confidential Disclosure Agreement (CDA) and a Material Transfer Agreement (MTA) to be signed. If approved by our Scientific Committee, these extractions of data can be used, analysed and results can be published under the supervision of the Scientific Committee and in association with the network coordinators.
