# Supplementary material for: Association Between Metabolic Syndrome, Obesity, and Cognitive Performances in Individuals With Bipolar Disorders: Cross‐Sectional and Longitudinal Analyses in the FACE‐BD Cohort
Source: Acta Psychiatr Scand. 2025 Nov 20;153(2):108–21. doi: 10.1111/acps.70048 (PMC12779237; doi:10.1111/acps.70048)
Supplement: Supplementary file 1 — FIGURE S1: Flowchart showing the selection of participants from the FACE‐BD cohort. FIGURE S2: Scree plot of Eigenvalues. TABLE S1: Result of the PCA: Eigenvalues of the correlation Matrix. TABLE S2: Results from PCA: Factor Pattern. TABLE S3: Association between global cognitive index and psychotropic medications in individuals with BD at baseline. TABLE S4: Association between global cognitive index, body mass index and MetS components. TABLE S5: Comparison between individuals with BD with a follow‐up visit and individuals with BD lost during follow‐up (univariate analysis). [file ACPS-153-108-s001.docx]

**Supplementary Figure 1: Flowchart showing the selection of participants from the FACE-BD cohort**

Individuals assessed at the first visit (V0) n=4853

Individuals with complete data to assess the metabolic syndrome criteria (presence or absence) n=3569

Euthymic individuals (MADRS <=10 and YMRS<=12), n=1924

**Exclusion criteria**

**No neuropsychological battery:** n=11

**Electroconvulsive therapy in the past year**: n= 295

**Neurodevelopmental disorder:**

- Dyslexia: 223
- Dysorthographia: 29
- Dyscalculia: 26
- Dysphasia: 3
- Dyspraxia: 14
- Language delay: 25
- Stuttering: 18

**Neurological disorders:**

- Stroke: 8
- Multiple sclerosis: 2
- Epilepsy: 19

**Substance-related disorders in the past month:**

- Alcohol consumption: 45
- Sedatives consumption: 4
- Cannabis consumption: 26
- Opioids consumption: 1
- Stimulants consumption: 0
- Cocaine consumption: 0

**Individuals who did not undergo neuropsychological testing**: 11

Number of selected patients n=1175

Individuals with metabolic syndrome: n= 252 (21.5%)

**Supplementary Table S1: Result of the PCA: Eigenvalues of the correlation Matrix**

| **Eigenvalues of the Correlation Matrix:**  **Total 14 Average = 1** | | | | |
| --- | --- | --- | --- | --- |
|  | **Eigenvalue** | **Difference** | **Proportion** | **Cumulative** |
| **1** | 6.09521393 | 3.64760015 | 0.4354 | 0.4354 |
| **2** | 2.44761378 | 1.46621731 | 0.1748 | 0.6102 |
| **3** | 0.98139647 | 0.09584107 | 0.0701 | 0.6803 |
| **4** | 0.88555541 | 0.05505090 | 0.0633 | 0.7436 |
| **5** | 0.83050451 | 0.22436149 | 0.0593 | 0.8029 |
| **6** | 0.60614302 | 0.11765769 | 0.0433 | 0.8462 |
| **7** | 0.48848534 | 0.09039367 | 0.0349 | 0.8811 |
| **8** | 0.39809167 | 0.04642456 | 0.0284 | 0.9095 |
| **9** | 0.35166711 | 0.02371442 | 0.0251 | 0.9346 |
| **10** | 0.32795269 | 0.07283973 | 0.0234 | 0.9580 |
| **11** | 0.25511296 | 0.10283974 | 0.0182 | 0.9763 |
| **12** | 0.15227322 | 0.03800980 | 0.0109 | 0.9871 |
| **13** | 0.11426342 | 0.04853695 | 0.0082 | 0.9953 |
| **14** | 0.06572647 |  | 0.0047 | 1.0000 |

**Supplementary Figure S2: Scree plot of Eigenvalues**

**
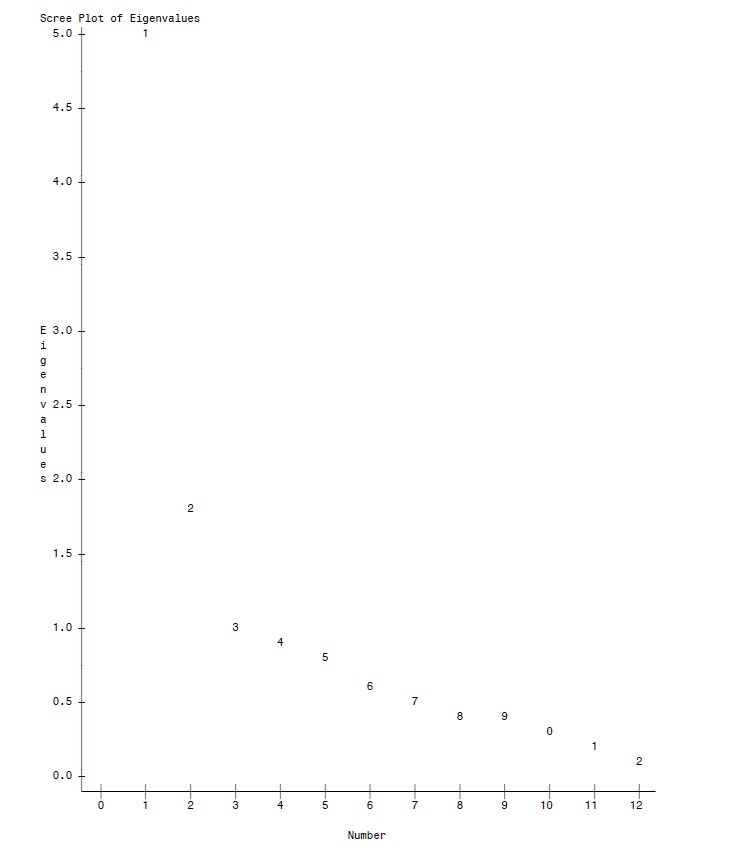
**

**Supplementary Table S2: Results from PCA: Factor Pattern**

| **Factor Pattern** | | |
| --- | --- | --- |
|  | **Factor1** | **Factor2** |
| WAIS-III/IV¹: digit symbol-coding (nb of correct symbols) | 0.64471 | -0.42882 |
| STROOP Test, Part C: nb of ink colors in 45s | 0.65870 | -0.43736 |
| TMT²-A: time in sec | -0.57312 | 0.43572 |
| TMT-B: time in sec | -0.63510 | 0.39844 |
| Verbal Fluency: nb of words with the letter P | 0.40821 | -0.25378 |
| Verbal Fluency: nb of animals named in 1 min | 0.54516 | -0.23857 |
| STROOP Test: Part A: nb of words read in 45s | 0.45398 | -0.51437 |
| STROOP Test: Part B: nb of colors named in 45s | 0.57143 | -0.48042 |
| CVLT⁴: nb of correct words | 0.81429 | 0.35137 |
| Short-delay free recall | 0.81450 | 0.46639 |
| Long-delay free recall | 0.82440 | 0.49406 |
| Short-delay cued recall | 0.80157 | 0.50634 |
| Long-delay cued recall | 0.81176 | 0.49238 |
| WAIS-III: Digit span subtest | 0.45803 | -0.15246 |

¹WAIS-III= Weschler Adult Intelligence Scale-III; ²TMT= Trail Making Test; ³CPT= Continuous Performance Task; ⁴CVLT= California Verbal Learning Test; nb = number

| **Treatment, mean (sd)** | **Global Cognitive Index,**  Estimated means (se) | Multivariable  P value* |
| --- | --- | --- |
| Antidepressants  Yes  No  Anxiolytics  Yes  No  Hypnotics  Yes  No  First generation antipsychotics  Yes  No  Atypical antipsychotics  Yes  No  Lithium  Yes  No  Anticonvulsant  Yes  No | 0.001 (0.05)  -0.11 (0.04)  -0.28 (0.07)  -0.03 (0.03)  -0.38 (0.09)  -0.04 (0.03)  -0.46 (0.11)  -0.04 (0.03)  -0.29 (0.04)  0.07 (0.04)  -0.20 (0.05)  -0.003 (0.04)  -0.05 (0.04)  -0.09 (0.04) | 0.24  0.001  0.0005  0.0002  <0.0001  0.001  0.43 |

**Supplementary Table S3: Association between global cognitive index and psychotropic medications in individuals with BD at baseline**

* Analysis of covariance adjusted on gender, age, and education level.

**Supplementary Table S4: Association between global cognitive index, body mass index and MetS components**

|  | **Global Cognitive Index,**  Estimated means (se) | Multivariable  P value* | Multivariable  P value** |
| --- | --- | --- | --- |
| High waist circumference  No  Yes  High blood pressure  No  Yes  Hypertriglyceridemia  No  Yes  High fasting glucose  No  Yes  Low HDL cholesterol  No  Yes  Overweight  No  Yes | 0.05 (0.04)  -0.08 (0.03)  0.008 (0.03)  -0.08 (0.05)  0.02 (0.03)  -0.18 (0.06)  -0.003 (0.03)  -0.17 (0.07)  0.03 (0.03)  -0.20 (0.05)  0.06 (0.04)  -0.12 (0.04) | 0.0234  0.1199  0.0028  0.0368  0.0001  0.0004 | 0.8110  0.1047  0.0141  0.3421  0.0127  0.0372 |

* Analysis of covariance adjusted on sex, age, and education level.

* Analysis of covariance adjusted on sex, age, education level, lifetime cannabis and alcohol use disorders, smoking status, sleep disorders, and current psychotropic medications (anxiolytics, hypnotics, antipsychotics, lithium, and anticonvulsant)

**Supplementary Table S5: Comparison between individuals with BD with a follow-up visit and individuals with BD lost during follow-up (univariate analysis)**

|  | Individuals lost during FU  N= 647 (55.1%) | Individuals at FU visit,  N= 528 (44.9%) | Univariate p value* |
| --- | --- | --- | --- |
| Sex, n (%)  Women  Men | 399 (61.7%)  248 (38.3%) | 329 (62.3%)  199 (37.7%) | 0.822 |
| Age, mean (sd) | 38.9 (13.3) | 40.8 (12.7) | 0.012 |
| Years of education, mean (sd) | 14.4 (2.7) | 14.6 (2.7) | 0.181 |
| Metabolic syndrome, n(%) | 147 (22.7%) | 105 (19.9%) | 0.239 |
| Global Cognitive Index, mean (sd) | -0.03 (1.02) | 0.04 (0.97) | 0.304 |
| BMI, mean (sd) | 25.8 (5.2) | 25.4 (5.0) | 0.464 |
| Cannabis use disorder, n (%) | 96 (15.9%) | 61 (12.2%) | 0.082 |
| Alcohol use disorder, n (%) | 110 (18.2%) | 66 (13.2%) | 0.024 |
| Anxiolytics use, n (%) | 79 (17.5%) | 77 (17.3%) | 0.933 |
| Lithium use, n(%) | 147 (32.6%) | 167 (37.5%) | 0.122 |
| SGA use, n(%) | 190 (42.1%) | 160 (36.0%) | 0.286 |
| Anticonvulsant use, n(%) | 235 (52.1%) | 231 (51.9%) | 0.953 |

^*^ χ² test for categorical variables and Student's or Mann-Whitney tests (depending on the distribution of the variables) for continuous variables; FU= follow-up; SGA: second generation antipsychotics
